# Hydro-physical and chemical suitability of rosewood sawdust as a hydroponic substrate under drip irrigation

**DOI:** 10.1371/journal.pone.0336497

**Published:** 2025-11-17

**Authors:** Smart Idumoro Samuel, Oluwaseun Temitope Faloye, Abiodun Afolabi Okunola, Adeolu Adediran, Viroon Kamchoom, Natdanai Sinsamutpadung

**Affiliations:** 1 Department of Agricultural and Biosystems Engineering, Landmark University, Omu Aran, Kwara State, Nigeria; 2 Department of Water Resources Management and Agrometeorology, Federal University, Oye-Ekiti, Ekiti State, Nigeria; 3 SDG 2 Food Security Research Group, Landmark University, Nigeria; 4 Materials Design and Structural Integrity Group, Department of Materials and Metallurgical Engineering, Federal University Oye-Ekiti, Ekiti State, Nigeria; 5 Department of Mechanical Engineering Science, University of Johannesburg, South Africa; 6 Excellent Centre for Green and Sustainable Infrastructure, School of Engineering, King Mongkut’s Institute of Technology Ladkrabang, Bangkok, Thailand; King George's Medical University, INDIA

## Abstract

Comprehensive characterization of hydroponic substrate is important to determine its suitability as a growing media. Therefore, the suitability hypothesis was tested by determining the rosewood sawdust substrate hydrological response at different sizes: < 0.425, 0.425–1.70 and 1.70–2.00 mm. The physical and chemical properties of the substrates were determined in the laboratory using standard procedures. Water storage and air capacity of the substrates were determined while regression models were developed for the water storage prediction with respect to the substrate sizes and pipeline distance away from the fertigation source. The highest total porosity of 75.92% was obtained for the large particle, while the lowest value of 72.57% was recorded in the finest particle and thus translated to improved moisture content and storage efficiency. The values of field capacity obtained for the coarse and fine particle were 133 and 159%, respectively. The developed regression model for the water storage produces coefficient of determination (R^2^) greater than 0.6 (60%), indicating a good prediction. Results showed that major nutrients required for plants growth, in the rosewood enhanced the nutrients (N, P, K, Mg, Ca) in the applied solution, and were mostly considered normal, when compared to the standard. However, the electrical conductivity of 31.2 mS cm^-1^ obtained in the substrate was too high, thus necessitating the need to pre-treat it for reduced electrical conductivity (EC) before use. Therefore, considering the enhancement in the nutrients solution when applied to the rosewood, the substrate is recommended for growing crops in hydroponics.

## 1. Introduction

The world population was projected to be around 8.6 and 9.8 billion by 2030 and 2050 respectively and that Nigeria, India and China will account for about 35% of this projected growth [1]. Despite program and other interventions aimed at increasing agricultural food production, Nigeria and other developing nations still struggles with food security [2]. The rising demand for food is exacerbated by the degradation of soil, the primary natural medium influencing root biomechanical properties, plant’s anchorage, crop growth, and the supply of air, water, and nutrients [3–7]. Similarly, some previous studies [8–11] emphasize the need to determine the component of the soil-less media to determine their suitability as hydroponics substance. Cultivating crops in hydroponics is important since it complements crops grown in soils, which are faced with degradation caused by factors, such as soil compaction, poor drainage, erosion, pest infestation, microorganisms and disease, etc. [9]. Compounding these issues, climate variations have been shown to significantly impact tree transpiration, further emphasizing the need for resilient agricultural practices [10,11]. To address these challenges and ensure food security, it is essential to adopt advanced, cost-effective systems of food production that reuse natural resources and increase agricultural productivity. Recent studies have highlighted the potential of soil additive to improve water retention and nutrient cycling in growing substrates [12–14]. Soil infertility, urbanization, climate change has been identified by a number of researchers [15,16], as the factors resulting in increased challenge in food insecurity. These researchers also indicated that these factors combine to hinder the optimal food production and is manifested as low yield, poor quality of food produce, reduced farmer’s income etc. Hydroponics emerges as a promising solution because it bypasses many soil-related limitations, allowing for controlled nutrient management and efficient water use, even in areas with poor or degraded soil [17]. By incorporating hydroponics system as an advanced technique to complement the use of soil in growing food crops, especially in areas where arable farmland is lacking, agricultural food production can be improved upon [18,19]. Soil-less farming was declared as a sustainable practice that has a more complementary solution to the limitations of soil farming and as a future for agricultural revolution that will encourage the youths into farming.

Hydroponics system is a soil-less, space saving, dirt-free and most importantly water effective technique or approach of growing crops. According to Sheikh [20], the substrate used as growing medium in this system helps to transmit the necessary nutrients in the irrigated nutrient solution and keeps the plant’s roots oxygenated. Soil-less farming was invented to complement the use of soil and to ensure food production where there are limited cultivable arable lands [21] and preferably carried out in a controlled environment. In terms of water usage, hydroponics or soil-less farming uses about 10% of water whereas, soil-based farming uses more than 80% of water [22]. Materials that are commonly used as growth media in hydroponics include coco-peat, sawdust, rock-wool, coco-coir, peat, vermiculite, perlite, pumice, rice husk, etc. [3] and among these aforementioned media, sawdust has been used for growing different types of crops in hydroponics, due to the fact that it has high carbon content, moderate water holding capacity, good aeration, good drainage capability, considerably cheap and locally available [23–26]. In addition, several studies [27–30] have considered the hydrological, and physical properties responses of substrates of different particle sizes, made from different sources, and reported differential impacts on the growth and yield of crops in hydroponically grown conditions. But very few studies have considered the impacts of sawdust made from rose wood at different particles sizes on the hydro-physical properties. The determination of this response is important for crop growth and yield determination of crops. Moreso, using sawdust as a potential growing medium may equally depend on the wood source and particle sizes, which may ultimately have combined effects on the growth, development and yield of the crop under hydroponic condition [27–30].

Rosewood (*Dalbergia latifolia*), a type of tropical hardwood timber which is highly prized for its durability, beauty and fragrance belongs to the legume family (*Fabaceae*). It is a native wood found in tropical and sub-tropical regions, particularly India, Southeast Asia, including Thailand, and in some Africa nation such as Nigeria [31]. In Nigeria, rosewood can be found in south-south Nigeria, southeast Nigeria, southwest Nigeria and in the rainforest region of Kwara State, i.e., Omu Aran, Nigeria. Omu Aran has a history of woodworking and sawmills specialized in processing woods (rosewood inclusive), which are sourced from local forests or imported from other parts of Nigeria [32,33]. Sawdust from rosewood may be considered as potential growth medium in Omu Aran locality due to its availability and unique properties such as fine texture, durability, low moisture content, etc.

Sawdust, a major waste of sawmill operation is basically disposed by conventional incineration (or burning), which often produce ashy materials and other hazardous substances such as carbon monoxide, sulphur oxides, nitrogen oxides, particulate emissions, etc., thereby causing environmental pollution [34]. Using sawdust for other beneficiary purpose becomes an urgent consideration as a unique way of solving the problems of waste disposal and hence, contributing to the systems of waste management [35]. The choice of sawdust as a test material in hydroponics may be related to its comparatively low cost and local availability, especially in Nigeria and other rain forest zones of Africa [36]. Research caried out by Dinkar *et al* [37], comparing physiological parameter values of three different substrates- sawdust, coco peat and sterilized absorbent cotton on crop growth recorded highest from the plants grown in sawdust which were in akin to the findings reported for cherry tomato grown in sawdust substrates [38–39] made from a tree called almond (*Terminalia ivorensis*). However, it is not understood if all sawdust produced from different trees can produce strong positive effects on the growth and yield of crops. For example [40], documented decline in the growth and yield of crops due to salinity and high electrical conductivity (EC) of some crops. Hence, this necessitates the need to comprehensively evaluate the physico-chemical and hydrological suitability of rosewood as a potential hydroponic substrate, due to its local availability in the study area and region. The consideration of sawdust of different particle sizes as growing media may be crucial, since particle size may affect some physical properties such as bulk density, total porosity, water retention ability and other hydrological properties of sawdust [36]. Apart from particle size of sawdust, other considerate physical properties which include low density, high porosity, high water retention, moderate water drainage, high bacterial tolerance and an acceptable rate of biodegradability may also offer some benefits on crops grown using sawdust under hydroponic condition [41–52]. The hydrological parameters obtained from the substrate may be affected by emitter discharge, drip operating pressure and distance away from the water source [53,54]. These parameters have all been observed to affect the hydrological efficiency parameters like the application efficiency and water retentions. Also, several authors posited that knowing the chemical characteristics such as nitrogen content, potassium, phosphorus, pH, electrical conductivity, etc., of a substrate are important before its usage as growing medium in hydroponics [36]. Sawdust has been commercially used for many years, but yet, data is lacking to verify if sawdust can be suitable as a standalone medium for growing of crops in hydroponics [4]. Therefore, the research aimed to characterize the physical, chemical and hydrological properties of different particle sizes of rosewood sawdust as potential growing media in hydroponic condition under drip irrigation, hence, filling the gap(s) where little has been done on sawdust using rosewood as a test crop, due to its domestic availability in the North central part of Nigeria.

## 2. Materials and methods

### 2.1. Study area

The study was conducted at Landmark University, Omu Aran, Nigeria. Omu Aran is a town in Southern Guinea Savannah, Nigeria’s sub-humid agro-ecological zone, situated at an elevation of 564 meters above mean sea level and at latitudes 8° 8′00′′N and longitude 5° 6′00′′ E [36]. It is the nearest town to the rainforest and derived savanna in Kwara State, in middle belt of Nigeria with tropical maritime climate, lengthy rainy season and mild weather all year long [55]. The average daily temperature falls between 16 and 32° C and depending on the difference in hot and cold weather during seasonal changes, the average rainfall depth is between 600 and 1200 mm, distributed across six to eight months of the year [55]. The experiment was carried out from January to August, 2024. A map describing the location of Omu Aran in Kwara State, Nigeria is shown in S1 Fig (supplementary material).

### 2.2. Experimental materials

The materials used in this study include rosewood sawdust, polyvinyl chloride (pvc) pipes and connectors, plastic bottles, reservoir/ tank, drip lines, liquid fertilizer (super gro), catch cans. stop clock, measuring cylinder, core sampler, electronic weighing balance, microprocessor pH meter, photometer, spectrometer and calorimeter.

### 2.3. Experimental methods

#### 2.3.1. Laboratory setup and procedure.

Rosewood sawdust was acquired from Adex Oni Asorted Woodwork industry, a sawmill located along Latiwon road in Omu Aran, Kwara State, Nigeria. Prior to collection, the milled wood was not given any special chemical treatment. The collected sawdust was air dried at room temperature, and taken to the soil and water laboratory at landmark university for further study.

**Fig 1:**
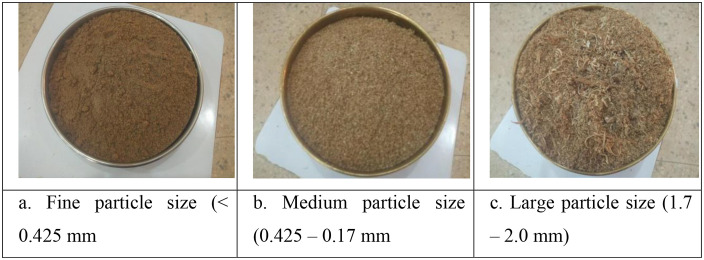
Different particle sizes of rosewood sawdust obtained from sieve analysis.

Seive analysis methods were used to separate the rosewood sawdust into distinct particle sizes. The collected sawdust was air dried and seived using a cleaned 2.00 mm diameter seive. Subsequently, sieves of diameters 0.425 and 1.70 mm were cleaned, assembled and used to separate the seived sawdust into three different particle sizes: fine (< 0.425 mm), medium (0.425–1.70 mm) and large (1.70–2.00 mm) as shown in Fig 1a–c). The samples of the different particle-sized sawdust were collected in similar cylindrical core samplers of 55 mm diameter and length, 100 mm in replicates of four (4) for the purpose of determining the physical and hydrological properties of the different particle-sized sawdust. Prior to this, the weights of the empty samplers were taken and recorded (W_i_). The weight of each sawdust in the core samplers were measured (W_1_) using an electronic weighing balance. To estimate the initial moisture content, the sawdust samples were oven dried for 12 hrs at 70 ^0^C [34]. The weight of the oven dried samples were measured (W_2_). The core samplers containing the different particle-sized sawdust were partially immersed in a dish containing water and allowed for 48 hrs, so as to get saturated by capillarity. The weights of the saturated samples were measured (W_s_) and then allowed to drain by gravity for 24 hrs. After-which, the final moist weights of the different particle-sized sawdust were taken (W_d_).

#### 2.3.1. Field experimental setup and design.

**Fig 2:**
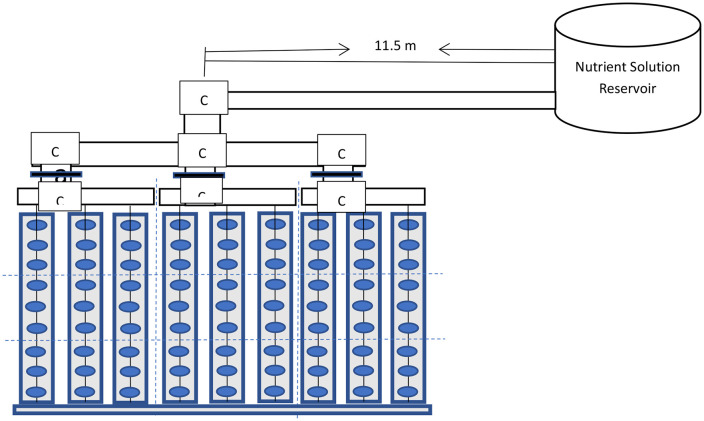
Schematic diagram of the drip fertigation system.

**Fig 3:**
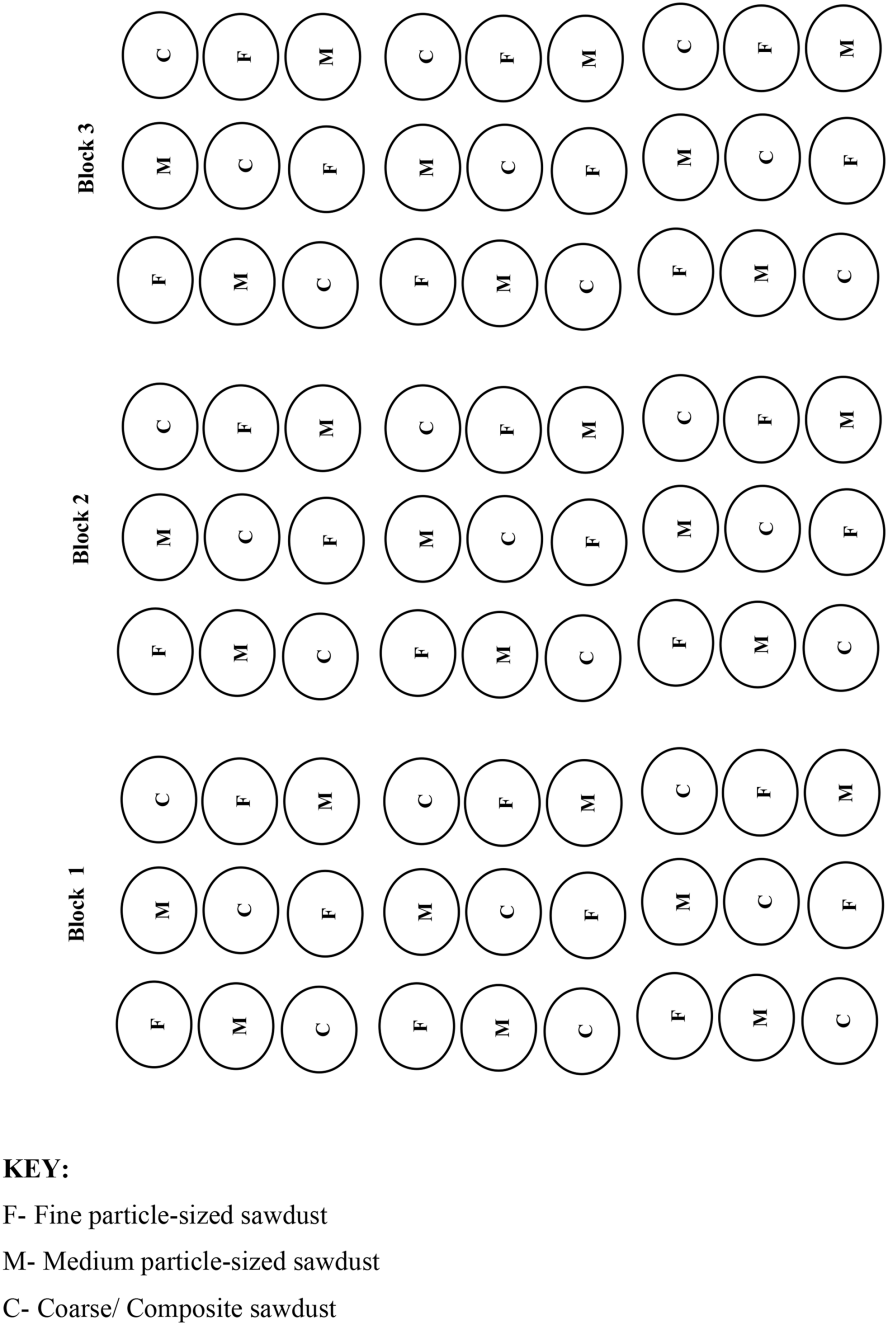
Experimental design/ arrangement of sawdust of different particle sizes for drip-fertigated irrigation.

The field experimental setup was done in a screen house in the teaching and research farms of the university. A gravity flow drip-fertgated irrigation system comprising of a 100 cm elevated tank/ reservoir, one inch (1“) main (pvc pipe), three sub-mains at three varied distances of 10, 11.5 and 13 m from the reservoir datum, three drip lines (rows) connected to each sub-main (pipe), each having 9 emitters that were 30 cm inter-spaced, to discharge the nutrient solution along each row. 9 of such four inches (4”) pvc pipes, with nine perforated holes 30 cm intra-spaced along each pipe. These were laid such that a drip-line ran over a pipe, with each emitter directed towards a hole along the pipe. Catch cans were inserted into the holes to collect the emitters’ discharges. Eighty-one (81) plastic bottles of diameter 7 cm and 10 cm long were perforated at the bottom to permit drainage were filled with the three distinct air-dried particle-sizes of rosewood sawdust and placed on catch cans in the various bored holes on each of the pipe. The over-head tank comprises of the mixture of liquid fertilizer and water. The liquid fertilizer used in the drip-fertigation was super-gro, with chemical compositions given on the label. Apart from carbon content and organic matter, all the other chemical elements measured for the rosewood sawdust were measured for the nutrient solution (1 litre of water to 1 ml of fertilizer) so as to validate the manufacturer’s label, using the various methods described for sawdust analysis. The experimental setup was made such that there were 27 sawdust stands per treatment. That is, three rows (pipes) each containing nine stands of sawdust of different particle sizes in a randomized complete block design (RCBD) for the three (3) levels of varied distances from the reservoir. The RCBD was employed in this study to take care of the heterogeneity of the different particle-sized sawdust and hence, to reduce bias or experimental errors (Figs 2 and 3)).

### 2.4. Experimental procedure and analysis

The physical and chemical analysis of the different particle-sized sawdust were carried out in the laboratory, standard methods were used to determine the chemical properties while the hydrological properties were estimated from the data collected in the field experimentation, using the methods/ formula described below. Phytochemical and other toxic elements present in the milled rose wood was removed by steaming process, that is, the sawdust was soaked in hot water that have been allowed to reach boiling point (100^o^C)

#### 2.4.1. Physical properties of rosewood sawdust.

The different **particle sizes** of the rosewood sawdust were determined from sieve analysis using sieves of different diameters to obtained: fine (< 0.425 mm), medium (0.425–1.70 mm) and large (1.70–2.00 mm).

The determination of **moisture content** was carried out by measuring the weight reduction between the initial/ air-dried weight and the weight of the sawdust subjected to oven drying at 70° C for 12 hours [34]. This lower temperature was maintained to avoid the pyrolysis or combustion of the substrate into ashes or charcoal, since at higher temperature the substrate could be extremely damaged as a result of heat [56]. The difference in weight gave the exact information on the moisture content, which was expressed as a percentage on dry basis using the expression of equation 1 [57].


$$\text{MC}=\frac{\left({\text{W}}_{\text{1}}\;-\;{\text{W}}_{\text{2}}\right)}{W_{\text{2}}}\;X\;\text{100}$$ (1)

where MC is percentage of moisture in the sawdust (%), W_1_ is the weight of the sawdust (g) at saturation and field capacity (after allowing the water to drain for 24 h) and W_2_ is oven-dried weight of sawdust (g).

The substrate bulk density was determined by standard method [58]. A standard sampling core was filled with substrate samples and compressed such that the substrate occupies the volume of the cylinder. The core lid was carefully closed and substrate was oven dried. The formula provided in equation 2 was used, which involves dividing the mass of dried sawdust by the total volume of sawdust or volume of the container containing the sawdust [57,58]. The determination of the bulk density was based on five replicates. The instrument used for the measurement of the mass was based on the resolution of 0.01 g, this was chosen to allow good precision.


$$\text{BD}=\frac{W_{\text{1}}}{\text{V}}$$ (2)

$$\begin{array}{c} V=\mathrm{\backslash sfrac}14\pi d^{\text{2}}h \end{array}$$ (3)

where BD is bulk density of sawdust (g cm^-3^), W_1_ is weight of dried sawdust (g), V is volume of sawdust/ container in cm^3^, d and h are the diameter and height of the cylindrical container respectively (cm).

The ratio of total pore volume to bulk volume is known as **total porosity**. In this study, total porosity was estimated using equation 4 [57].


$${\text{P}}_{\text{T}}=(\frac{V_{w}}{V_{T}})\;X\;\text{100}$$ (4)

where P_T_ is the total porosity in percent, V_w_ is volume of water in the sawdust (cm^3^) and V_T_ is the volume of the sawdust/ container containing the sawdust (cm^3^).

#### .2.4.2. Chemical properties of rosewood sawdust.

A microprocessor pH meter was used to measure the pH of the sawdust that had been collected. After being air-dried, 25 g of the sawdust was sieved using a 1.70 mm mesh size and then put into a 100 ml beaker. After adding 5 cm^3^ of distilled water, the mixture was vigorously agitated with a glass rod for 30 minutes. A standardized pH meter’s electrode was placed into the mixture, and the digital reading was used to determine the pH in the solution [59]. Using the 1:2 dilution procedure, the EC was determined. Following the HCl digestion of the sample ash, nutrients were analyzed using the Kjeldahl method to determine total nitrogen (N). For the total nitrogen determination, 1 gram of the substrate sample was weighed and placed in kjeldahl flask. A digestion catalyst (selenium) was added into the flask with 10 ml of concentration of HCL added. The distillate was collected in 50 ml of 0.1 M HCL. Nutrients were measured on a filtered extract using a weight equivalent to 60 ml of the sample and 300 ml of deionised water [60]. Photometric analysis was used to identify potassium (K), sodium (Na), and calcium (Ca). The concentrations of phosphorus (p) and sulphur (S) were measured by colorimetric method, boron (B) and chlorine were determined by titration. CEC (cation exchangeable capacity) and SAR (sodium absorption ratio) were estimated from the measured positive ions (cations) values, using the equations 5 and 6 respectively [58]. The SAR was calculated to assess the risk of salt accumulation in the substrate and plant


$$\text{CEC}=\frac{sum\;of\;all\;positive\;ions\;(cations)}{Mass\;of\;substance}$$ (5)

$$\text{SAR}=\;\frac{{\text{Na}}^{+}}{\sqrt{}\frac{{\text{Ca}}^{\text{2}+\;}+\;{\text{Mg}}^{\text{2}+}}{\text{2}}}$$ (6)

#### 2.4.3. Hydrological properties of different particle-sized sawdust.

The time to run the operation of the drip-fertigated irrigation system in this study (irrigation time) was established in the laboratory using equation 7 [61], and was estimated to be 2 days interval.


$${\text{t}}_{\text{i}}=\frac{\text{d}\;\text{x}\;\text{A}}{\text{Q}}$$ (7)

where t_i_ is irrigation time (mins), d is irrigation depth (cm), A is the total area to be watered (cm^2^) and Q is the discharge (L min^-1^). The emitter discharges nutrient solution at 0.4 L h^-1^

The drip irrigation system was run on the different particle-sized sawdust completely randomized in the setup for the period of estimated time (t_i_). The weights of the saturated sawdust were taken and the surfaces were covered with thick cover (thick polythene made material) to prevent moisture lost to evaporation and allowed to drain by gravity for 24 hrs. They were then oven dried for 12 hrs at 70 ^0^C and the weights were equally taken. The hydrological properties of the different particle-sized rosewood were estimated using equations 8–11 [45,62].


$${\text{MC}}_{\text{s}\;}=\frac{{\text{W}}_{\text{s}}-{\text{W}}_{\text{2}}}{{\text{W}}_{\text{2}}}\times \text{100}$$ (8)

$${\text{MC}}_{\text{d}\;}=\frac{{\text{W}}_{\text{d}}-{\text{W}}_{\text{2}}}{{\text{W}}_{\text{2}}}\times \text{100}$$ (9)

$$\text{AC}={\text{MC}}_{\text{s}}-{\text{MC}}_{\text{d}}$$ (10)

$$E_{s}=\frac{W_{R}}{W_{A}}\;\times \;\text{100}$$ (11)

where MC_s_ is the percentage moisture content at saturation (or water storage capacity), MC_d_ is the percentage moisture content after drainage,W_s_ is the saturated weight of sawdust (g), W_d_ is the weight of moist sawdust after drainage (g), W_2_ is the oven dried weight of the sawdust (g), AC is the percentage air capacity, E_s_ is the storage efficiency (%), W_R_ is the amount of water retained by the substrate (usually after 24 hours) and W_A_ is the amount of water applied.

### 2.5. Statistical analysis

The mean values of the physical and hydrological characteristics of the different particle-sized rosewood sawdust: bulk density, moisture contents, total porosity, air capacity, storage efficiency were subjected to one–way ANOVA (analysis of variance) using Minitab version 17 and were compared using Tukey method at 95% confidence level to determine their significant difference. Regression models were developed using Minitab version 17 for the various hydrological parameters in relation to particle size and irrigation distance, and the model was evaluated using the coefficient of determination (r^2^), which has been commonly and generally used in evaluating models in engineering and sciences [63–69]. Also, a quadratic surface response plots for the hydrological properties were developed to explain their relationships with respect to particle size and irrigation distance. The quadratic (non-linear) relationship has also been used to show relationship between input and output by a number of researchers [70–74]

## 3. Results and discussions

### 3.1. Physical properties of rosewood sawdust; particle analysis, bulk density and total porosity

The different particle sizes of the rosewood sawdust were determined by seive analysis into fine particle size (< 0.425 mm), medium particle size (0.425 －1.70 mm) and large particle size (1.70 － 2.00 mm). A substrate’s bulk density plays a crucial role in deciding whether or not it is suitable for use as a growing medium in a hydroponics system. According to the report of Oludare [75], sawdust used in hydroponics has an optimal bulk density that falls between 0.10 to 0.30 g cm^-3^. This range allows for good aeration in the root zone, adequate water holding capacity and healthy root growth and development. According to Wang [74], sawdust whose bulk density falls within 0.15–0.25 g cm^-3^ were ideal for proper aeration, root development and optimal growth of plants. Sawdust with a bulk density of 0.18 to 0.22 g cm^-3^ was found to enhance plant output and water retaining capacity in the study by Oludare et al. [75]. The values obtained in this study were considered as low bulk densities and fall within the ideal range for hydroponic substrate: fine, medium and large particle-sized sawdust were 0.282, 0.259 and 0.241 g cm^-3^ respectively as shown in Table 1. However, the difference in their mean values were statistically significant (p > 0.05). This may be due to the fact that the smaller particles are more tightly packed together thereby, leaving less empty space between the sawdust material. Although, low bulk density media might not offer enough support for plant anchorage, but according to Nagaraj et al. [58], they are better in mixing and transporting than high bulk density substrates. Moreso, the bulk density obtained in this study is within the range reported ideal (< 0.4 g cm^-3^) for hydroponic substrates [76,77]. Theses researchers reported that this range of bulk density support the growth and development of plants under hydroponic conditions. Hence, sawdust used as substrate in growing crops hydroponically may require irrigated shade house frequently to avoid oxygen depletion. The bulk density value obtained in this study is dependent on the sizes and possible microstructure of the substrates, which also affect the water retention properties and other physical and mechanical properties of the substrates [78–80].

**Table 1:** Physical properties of Rosewood sawdust of different particle sizes.

Description	BD (g/cm^3^) (± SE)	P_T_ (%) (± SE)
**F**	0.2819^a^ (± 0.0015)	72.57^a^ (± 0.79)
**M**	0.2594^b^ (± 0.0028)	74.17^a^ (± 1.53)
**L**	0.2408^c^ (± 0.0023)	75.92^a^ (± 1.99)

Note: SE is the standard error; the means with the same letter(s) in the same column do not differ significantly (p ≤ 0.05); F is fine sized sawdust, M is medium sized sawdust and L is large sized sawdust, BD is bulk density and PT is total porosity.

The total of the pores that are filled with air and water is known as total porosity. It is necessary for the best possible plant growth and has a balanced ratio for both water and air capability. Total porosity of sawdust may range from 70–85%, as beneficial for plant growth, allowing adequate water to be held, good aeration and healthy root growth and development [81]. According to Wever [82], too high porosity may lead to increased drainage and risk of root drying off, while too low porosity on the other hand, may result to poor aeration, increased risk of root rot and waterlogged condition. In this study, the total porosity of rosewood sawdust increases with increasing particle sizes, i.e., 72.57, 74.17 and 75.92% for fine, medium and large particle-sized sawdust respectively (Table 1), and fall within the range ideal for plant growth [81]. The differences in the mean values of total porosity obtained are statistically insignificant at 95% confidence level. These values falling within the recommended aforementioned range for optimal plant growth and development, implies that sawdust-use as a potential growing medium in hydroponics will promote aeration of plant’s roots. Identifying the best particle size for crop growth is important for optimizing productivity and also beneficial to improved farmer’s income. Some researchers across the globe have also confirmed and acknowledge optimization process as a means of enhancing economic profitability [83–88].

There was an inverse proportionality between the bulk density and total porosity in relation to the different particle-sized rosewood sawdust. That is, with reducing particle size, bulk density increases whereas total porosity decreases.

### 3.2. Chemical properties of rosewood sawdust and solution nutrient in substrate

The proper growth and development of plants grown in hydroponic systems depends on the chemical components of the substrates used. According to Jones [89], these components boost plants’ defence against pests and diseases, optimize agricultural yields and nutritional value and preserve ecosystem balance and fertility. The chemical compositions of rosewood sawdust measured are presented in Table 2, alongside with the range recommended for substrates as growth media in hydroponics.

**Table 2:** Chemical characteristics of rosewood and the recommended range of values for substrates used in hydroponics.

Element	Symbol	Substrate	Recommended Value	Nutrient solution applied	Nutrient mixed with substrate	Recommended values (mmol L^-1^)
Nitrogen	N (%)	1.24	0.5-3.5^a^	8.1 ± 0.50	8.8 ± 0.48	–
Phosphorus	P (mmol L^-1^)	0.55	NA	0.54 ± 0.30	0.84 ± 0.25	0.5 – 1.5^b^
Potassium	K (mmol L^-1^)	0.74	NA	4.57 ± 0.63	5.2 ± 0.51	4–8^[94]^
Calcium	Ca (mmol L^-1^)	0.55	NA	3.60 ± 0.50	4.1 ± 0.52	3–8^[94]^
Magnesium	Mg (mmol L^-1^)	0.54	NA	2.5 ± 0.23	3.0 ± 0.28	2–5^[94]^
iron	Fe (mmol L^-1^)	0.03	NA	1.0 ± 0.26	0.75 ± 0.22	2.5 - 5.0^[94]^
Manganese	Mn (mmol L^-1^)	0.011	NA	0.01 ± 0.00	0.014 ± 0.00	5^[94]^
Copper	Cu (mmol L^-1^)	0.025	NA	0.34 ± 0.11	0.44 ± 0.09	1^[94]^
Zinc	Zn (mmol L^-1^)	0.015	NA	0.32 ± 0.14	0.45 ± 0.12	5^[94]^
Boron	B (mmol L^-1^)	0.082	NA	0.53 ± 0.15	0.75 ± 0.17	30^[94]^
Sodium	Na (mmol L^-1^)	0.074	NA	0.52 ± 0.18	1.4 ± 0.16	<3-5^[94]^
Potentials of hydrogen	pH	6.39	5.2–6.3^[77]^		6.34 ± 0.15	5.5 – 7.3^a^
Electrical conductivity	EC (mS cm^-1^)	31.2	<0.5^[77]^		6.8 ± 0.11	1.4–4^94^
Organic matter		16.15				–
Carbon	C (%)	64	NA		–	–
C/N	C/N	51.6	10-80^[a]^		–	–
Cation exchangeable capability	CEC (meq L^-1^)	10.6	10 – 20^b^		–	–
Sodium absorption ratio	SAR	0.98	< 1		–	–

Note: N-S means nutrient solution in substrate ^a^[95], ^b^[94]; NA is not available

#### 3.2.1. Nitrogen (N).

An essential ingredient that supports plant growth, leaf production, and stem development is nitrogen. Nitrogen is a highly mobile element in plants and is necessary for the synthesis of proteins, nucleic acids, and other cellular components that are necessary for all life forms. The study of Marschner [90] posited that, excess Nitrogen may result to leaf burning, while shortage of it may cause stunted growth and yellowing leaves. In this study, the nitrogen content measured for rosewood sawdust was 1.24% which falls within the recommended range of value (0.5–3.5%) based on standard (Table 2). Also, a total nitrogen content of 8.8% was observed in the nutrient solution when mixed with the substrate. This enhanced total nitrogen value can be attributed to the additive effects of the rosewood nitrogen with the applied nitrogen (8.1%). Frequent supply of drip-fertigated solution that is rich in nitrogen, may help to improve the nitrogen content in sawdust and hence, may ensure proper growth and development of crop grown in sawdust.

#### 3.2.2. Phosphorus (P).

This chemical element supports root development, flower and fruit formation, and overall plant energy. A study by Havlin et al. [91] claims that an abundance of phosphorus might hinder root growth and decrease the uptake of micro-nutrients, whilst a deficiency can cause stunted growth and decreased root development in plants. In this study, the rosewood sawdust was measured and the value of phosphorus was found to be 0.55 m mol L^-1^. The nutrient solution in the substrate gave a value of 0.84 m mol L^-1^ which was considered sufficient for plant growth in hydroponics, when compared to the recommended range (0.5–1.5 mmol L^-1^) based on standard. Supplying phosphorus through effective drip-fertigated irrigation system may increase the concentration of phosphorus in the sawdust as a growing medium from deficiency to optimal level and hence, improving the vegetative and reproductive parts which will enhance improved crop quality. Similar to the observation in the nitrogen level, the substrate enhanced the phosphorus content as well, when nutrient solution was added to the substrate.

#### 3.2.3. Potassium (K).

Potassium is a chemical element that helps with overall plant health, resistance to disease and water balance. Excess of it may reduce plant’s uptake of calcium and magnesium and thus, causes leaf scorch, while shortage of potassium supplied to plants may result into reduced growth and yellowish of leaves [91]. In this study, the rosewood sawdust measured has potassium value of 0.74 mmol L^-1^. The nutrient solution in the substrate produced a value of 5.2 mmol L^-1^, which is within the normal range of values reported in Table 2 for potassium. The use of the right substrate like rosewood could help avoid stunted growth in hydroponics, through the enhancement of the macro-nutrient like Potassium, particularly when nutrient solution is added

#### 3.2.4. Magnesium (Mg).

Magnesium is essential in chlorophyll development, for photosynthesis, cell wall development, and nutrient uptake. Previous studies reviewed that reduced manganese uptake and burnt leaf tip in plant was as a result of excess magnesium application while reduced growth and yellowish leaves was caused by shortage of magnesium supplied to the plants [91]. The recommended magnesium requirement for plants growing on soil-less medium should be between 2–5 mmol L^-1^ (Table 2). This study valued the magnesium content in rosewood sawdust to be 0.54 mmol L^-1^. The magnesium content in nutrient solution falls within the recommended range (Table 2). Thus, there may be need for frequent irrigate of sawdust used as growing medium in hydroponics. Using a drip-fertigated irrigation system whose nutrient solution is rich in magnesium will help to avoid reduced growth and yellowing leaves of plants.

Overall, the high essential macro-nutrients obtained in this study could be attributed to the high-water retention and porosity value reported in this study, which resulted to improved nutrient retention from the fertigation, and also the type of rosewood used which resulted to enhanced nutrient availability.

#### 3.2.5. Sodium (Na).

Sodium may not be essential for plants, but can be tolerated in small amounts, usually less than 20 ppm. If not managed, excessive sodium supply to substrate may result to reduced plant growth and ion imbalance. The measured value of sodium (0.074 mmol L^-1^) in this study, indicates the considerate amount of sodium in the rosewood sawdust. The accumulation of sodium beyond the allowable limit could lead to high salinity and SAR, which could prompt the decline in growth and development of plant due to osmotic pressure. The lower Na and SAR values obtained in this study ascertained the suitability of the nutrient and the rosewood substrate in cultivating crops under hydroponic conditions.

#### 3.2.6. Calcium (Ca).

Calcium is a crucial chemical element that is useful for the development of plant’s cell wall, root growth and nutrient uptake. There is no established value for calcium substrate for hydroponics condition. Based on laboratory measurements, calcium content obtained in the measured rosewood sawdust was found to be 0.55 mmol L^-1^, which can also serve as a reference for other and further study, for comprehensive comparison. Compared to the recommended range, and as such, the calcium content in the nutrient solution of the substrate falls within the standard range (Table 2). According to the study of Marschner [90], excess of calcium in a substrate may cause reduced iron and manganese uptake by plant and might result into burnt leaf-tip, while shortage of it may cause reduced growth and blossom end rot.

#### 3.2.7. Boron (B).

Boron is a micro element that may be needful to plant for sugar metabolism, development of cell wall and nutrient uptake. Deficiency of boron in substrate may cause poor fruiting, leaf deformation etc., while excess of it may may results into reduced growth and burnt leaf-tip [91]. In this study, the measured amount of boron in rosewood sawdust was 0.082 mmol L^-1^ while the nutrient solution gave a value of 0.75, which was lower than the upper limit of 30 mmol L^-1^ reported in Table 2, based on standard.

#### 3.2.8. Iron (Fe).

Iron is essential for photosynthesis, respiration, and enzyme function. The study of Marschner [90], reported that excess of iron in substrate may cause reduced growth and leaf bronzing, while iron deficiency may result into chlorosis and poor growth. The value of iron measured in the rosewood sawdust was 0.03 mmol L^-1^. The nutrient solution in the rosewood substrate produced a value of 0.75 mmol L^-1^, which is lower than the value reported based on standard in Table 2.

#### 3.2.9. Manganese (Mn).

It is a micro element that supports photosynthesis, enzyme function, and plant defense. The study of Marschner [90], reported that excess of shortage of manganese in a growing medium can lead to chlorosis and reduced growth in plants. The measured value of manganese in rosewood sawdust in this study was 0.011 mmol L^-1^, while the nutrient solution produced a value of 0.014 mmol L^-1^ which is lower than the upper limit of 5 mmol L^-1^ reported based on standard in hydroponics condition. Thus, this value meets the optimal requirement for manganese using sawdust as the growing medium in hydroponics.

#### 3.2.10. Copper (Cu).

Table 2 revealed the copper concentration of rosewood as a substrate under hydroponic condition. The study by Marschner [90] reported that, copper is essential for enzyme function and disease resistance and that deficiency of copper in substrate used as growing medium can cause toxicity, poor growth, increased susceptibility to disease and yellowing leaves. The measured value of copper in the substrate nutrient solution produced a value of 0.44 mmol L^-1^, which is lower than a value of 1 mmol L^-1^ reported based on standard. Therefore, based on this copper content value, the rosewood could be considered as a suitable substrate under hydroponics condition.

Overall, the lower values of the heavy metals obtained in the study, the rosewood substrate, make it a potentially good substrate to be considered as hydroponics substrate. This is because too high values of heavy metals in a particular substrate or growing medium, could make human susceptible or prone to diseases, such as liver and kidney injury, hence the need for comprehensive assessment of the nutrients in both the rosewood sawdust and its solution.

#### 3.2.11. Organic matter and carbon contents.

The measurement of organic matter and carbon contents of rosewood sawdust were relevant because these elements impact the flow and mobility of nutrients for the benefit of micro-organisms, improvement of substrate’s structure and water-holding capacity, enhancement of cation exchangeable capacity and support plants’ development and growth [89]. Table 2 shows the values of the measured organic matter and carbon content in the rosewood sawdust as 16.15% and 64% respectively. The value of the measured carbon content was higher than the recommended range (10–80%) reported based on standard (Table 2). Excess carbon content may lead to rapid microbial growth, pH and nutrient imbalance, retention of excess water which may lead to waterlogged conditions [76] and hence, irrigation need to be frequent and moderate.

In addition to all the nutrients measured, the C/N ratio estimated from the carbon and Nitrogen contents produced a normal range (Table 2). The value of 51.6 fall within the standard range reported in Table 2. This shows that the substrate would enable the release of nutrient including nitrogen to the plant

#### 3.2.12. Potentials of hydrogen (pH).

The pH of any substrate is a measure of the relative acidity or alkalinity of the substrate. According to Callie [92], a lower value indicates a higher concentration of hydrogen ions and a lower pH. Generally speaking, the pH range obtained for both substrate and the nutrient solution compare favourably well with standard values (Table 2), and are considered ideal for crop growth and development [92]. A pH value outside of this range might cause the crop to starve or overdose on a particular nutrient, which can also result in decreased growth and production, toxicities or nutrient shortages, decreased water uptake, and heightened disease susceptibility [4,93]. The pH value of 6.39, which was measured in this study, is within the range that is ideal for plant growth and development. Regular drip-fertigated irrigation with nutrient solution below neutral pH, may help to regulate or/and maintain the pH of the sawdust used as growing medium in a hydroponics condition.

#### 3.2.13. Electrical Conductivity (EC).

In any substrate, the total amount of dissolved salts, also known as free ions, and their concentration are measured by the substrate’s EC. Excessive salt can cause the growing substrate’s osmotic potential to be severely impacted, which can lead to water loss and stress on the roots of the plants [92]. Too high a value can burn nutrients, while too low may limit plant’s growth [93]. In this study the EC of rosewood sawdust measured was 31.2 mS cm^-1^, which was considered too high compared to the recommended range of < 4 mS cm^-1^ reported based on standard (Table 2) for substrates. This lower value in the nutrient solution added, resulted in reduced EC value of (6.8 mS cm^-1^). This value was slightly higher than the standard range between 1.4 and 4 mS cm^-1^ (Table 2) reported for nutrient solution in substrates [94–96]. This implies that when the right nutrient solution with lower EC is applied, the substrate becomes more suitable for use in hydroponics.

#### 3.2.14. Cation exchangeable capacity (CEC).

Exchangeable cations, or positively-charged nutritional ions like Ca, Mg, K, and NH_4_, are measured by CEC, which is a substrate’s capacity to absorb and retain them. According to Callie [92], a substrate’s CEC measurement indicates its ability to hold onto and release more nutrients for plant growth and development. It also indicates the substrate’s resilience to leaching while watering. A higher CEC indicates that the growing medium is able to both absorb and release cations from the nutrient solution, which helps to stabilize the pH and nutrient concentrations surrounding the root zone. In this study, the value of CEC of the rosewood sawdust measured was 10.6 meq L^-1^ and this falls within the recommended range for substrates used in hydroponics (10–20 meg L^-1^). Drip-fertigated irrigation system will help to maintain the balance of cation exchangeable capacity of sawdust used as growing medium in hydroponics.

#### 3.2.15. Sodium absorption ratio (SAR).

SAR affects the sodium uptake and water infiltration through the growing medium. In this study, the rosewood sawdust was estimated to have SAR value of 0.98, which is less than one (< 1), the recommended range for substrates used in hydroponics. Using drip-fertigated irrigation to supply nutrient solution to sawdust may help to maintain balance of sodium absorption ratio of the rosewood sawdust as growth medium used in hydroponics. Table 2 gives detailed measured values of the chemical compositions of rosewood sawdust and the recommended values for hydroponic substrates.

### 3.3. Hydrological properties of rosewood sawdust of different particle sizes

#### 3.3.1. Moisture content.

The mean moisture content (MC) at saturation in this study were 301, 294 and 291% for fine, medium and large particle-sized rosewood sawdust respectively (Table 3). Statistically, there was a significant difference between the means of the fine and large particle sizes (p > 0.05), while the difference between their means and that of the medium particle were statistically insignificant at 95% confidence level. This shows that, “the finer the particle-sized sawdust, the higher the moisture absorbed”. However, the three distinct particle sizes of rosewood sawdust in this study were considered to have good water absorbing capability and should be considered as a potential growing media in a hydroponics condition. The high-water retention obtained in this study is similar to that of Mariyappillai and Arumugam [97] who reported a value in the range of 47.18–769.30% for various substrate, with a value of 369.03% reported for sawdust, which similar to those obtained in our study. However, lower values in the range of 23.2–71.8% were reported by Marinou et al. [32] due to larger particle sizes in the range of 4–8 mm used in their study, which is much lower than the range used in our study. These researchers reported enhanced growth of plants due to the higher water retention

**Table 3:** Hydrological characteristics of rosewood sawdust of different particle sizes.

	Moisture Content (%)		Air capacity,	Storage efficiency
Size	at saturation (± SE)	Field capacity -Drained (± SE)	AC (%) (± SE)	E_S_ (%) (± SE)
**F**	301^a^ (± 1.68)	159^a^ (± 2.27)	142^b^ (± 0.91)	52.82^a^ (± 0.49)
**M**	294^ab^ (± 2.80)	142^b^ (± 1.08)	152^a^ (± 2.00)	48.30^b^ (± 0.26)
**L**	291^b^ (± 1.58)	133^c^ (± 1.96)	158^a^ (± 2.27)	45.71^c^ (± 0.67)

SE is standard error; means with the same letter(s) in the same column do not differ significantly (p ≤ 0.05); F is fine sized sawdust, M is medium sized sawdust, L is large sized sawdust.

After drainage, the mean MC in the fine, medium and large particle-sized sawdust were found to be 159, 142 and 133% respectively (Table 3). The difference in their mean values were statistically significant (p > 0.05). Like at saturation, the three distinct particles had good water retaining capability. But the fine particle sized sawdust retained most when compared with the other retaining ability.

#### 3.3.2. Air capacity.

The air capacity (AC), which is also known as aeration porosity is referred to as the ability of a substrate to hold and transmit air to plant root. AC is crucial for healthy root growth and plant development [71]. The large particle sized sawdust has the highest (AC equals 158%), followed by medium (AC equals 152%) and least was the fine particle-sized sawdust (AC equals 142%) as described in Table 3. The difference in the mean values of medium and large particle-sized sawdust were statistically insignificant at 95% confidence level. However, there was significant difference between the aforementioned means and the mean of the fine particle-sized sawdust (p > 0.05). The difference could be related to the varied pore sizes in the different particle-sized sawdust. Generally, the air capacity of the three distinct particle sizes of rosewood sawdust are considerate and will allow good aeration in the crop root zone when used as growing media in hydroponic conditions.

#### 3.3.3. Storage efficiency.

The term “storage efficiency” describes sawdust’s capacity to hold and release nutrients and water when utilized in hydroponic gardening. It is associated with the sawdust’s bulk density and packing effectiveness. The storage efficiency in a substrate is influenced by moisture content, particle size and compaction [81]. In this study, the storage efficiency was found to be highest in the rosewood sawdust of fine particle size (52.82%), followed by the medium particle-sized (48.30%) and least in the large particle-sized sawdust (45.71%) as shown in Table 3. These estimated values show that the three distinct particle sizes of rosewood sawdust have good storage efficiency of moisture and should be considered as potential growing media in hydroponic condition. However, there difference in these mean values are statistically significant (p < 0.05) at 95% confidence level. The difference in moisture storage capability can be related to the difference in particle sizes, total porosity, bulk density, etc.

### 3.4. Modelling of main and Interactive Effects of hydrological Characteristics of Rosewood Sawdust in relationship to Particle Sizes and Irrigation Distance

The main and interactive effects of the parameter of the hydrological properties of rosewood sawdust (moisture content, air capacity and storage efficiency) were analyzed. The interactive effects of these parameters in relationship to the different particle sizes and irrigation distance from the reservoir were statistically insignificant at 95% confidence interval level. The explanatory linear regression interaction and full quadratic models for these parameters in relation to the particle sizes and distance from the reservoir are presented in Table 4. With higher R^2^ values (i.e., greater than 50%) in each of the parameters, the prediction of the hydrological parameters was higher with the two models. The full quadratic model gave the better predictions in each case, having higher values of coefficient of determination (R-square), indicating that the values of the variability in the response of the hydrological parameters on particle size and distance could be better explained by their full quadratic models.

**Table 4:** Regression models for hydrological properties of rosewood sawdust in relation to particle size and irrigation distance.

Parameter	Regression models	Equation	R^2^ value
Moisture content at saturation (MC_A_)	Linear regression + interaction Full quadratic model	MC_A _= 3.889 + 0.152 S^ns^ - 0.0670 D^ns*^ − 0.0214 SD^*^ MC_A_ = 1.23 + 0.465 S^ns^ + 0.380 D^ns^ - 0.1336 SS^ns^ - 0.0194 DD^ns^ - 0.0214 SD^*^	65.29% 68.64%
Moisture content after drainage (MC_B_)	Linear regression + interaction Full quadratic model	MC_B_ = 1.692 - 0.090 S^ns^ - 0.0049 D^*^ − 0.00260 SD^*^ MC_B_ = 2.353 + 0.164 S^ns^ - 0.137 D^*^ − 0.1083 SS^ns^ + 0.00577 DD^*^ − 0.00260 SD^*^	62.48% 65.96%
Air capacity (AC)	Linear regression + interaction Full quadratic model	AC = 2.197 + 0.242 S^ns^ - 0.0621 D^ns^ - 0.0188 SD^*^ AC = −1.12 + 0.301 S^*^ + 0.517 D^ns^ - 0.0252 SS^*^ − 0.02519 DD^ns^ - 0.0188 SD^*^	60.95% 64.63%
Storage efficiency (E_S_)	Linear regression + interaction Full quadratic model	E_S_ = 5.937 - 0.314 S^ns^ - 0.0171 D^*^ − 0.0091 SD^*^ E_S_ = 8.25 + 0.575 S^ns^ - 0.482 D^*^ − 0.380 SS^ns^ + 0.0202 DD^*^ − 0.0091 SD^*^	62.48% 65.96%

ns indicates insignificance, while * indicates significance at p ≤ 0.05 level of significance.

The R^2^ values given by the full quadratic models 68.64% (MC at saturation), 65.96% (MC after 24 hours), 64.63% (AC) and 65.96% (E_S_) entail higher predictions with the variables: particle size (S) and distance (D). The remaining percentage/ fractions of for the parameters, which are 31.36% (MC at saturation), 34.04% (MC after 24 hours), 35.37% (AC) and 34.04% (E_S_) can be due to other extraneous factors in the study that are neither size nor distance. The r-square values obtained in the study is also equivalent to coefficient of correlation (r) of approximately 0.8, which was considered moderately strong [97,98]

### 3.5. Surface response of moisture content, air capacity and storage efficiency with respect to particle sizes of rosewood sawdust and irrigation distance

**Fig 4:**
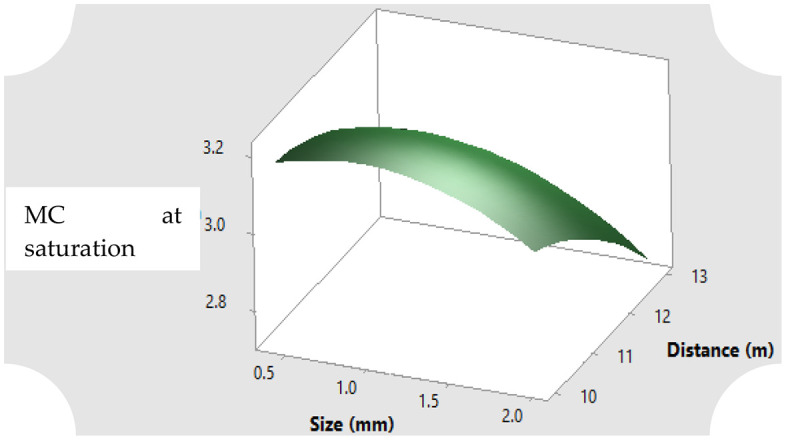
MC at saturation response of the rosewood substrate to different particle sizes and distance away from fertigation source.

**Fig 5:**
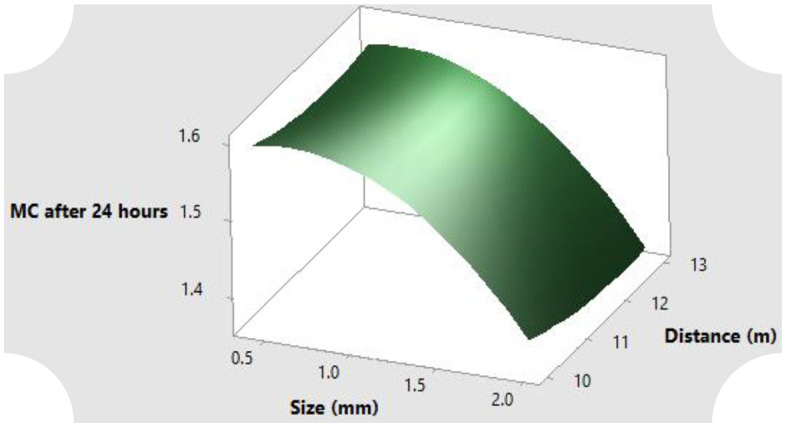
MC after drainage response of the rosewood substrate to different particle sizes and distance away from fertigation source.

**Fig 6:**
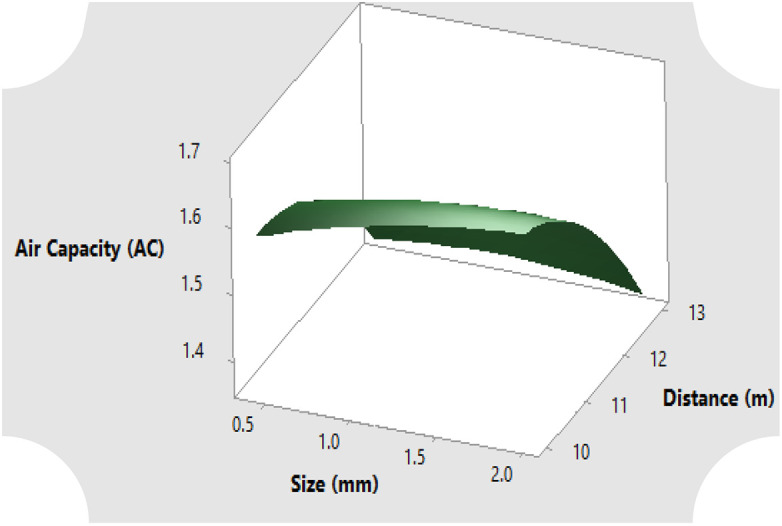
Air capacity response of the rosewood substrate to different particle sizes and distance away from fertigation source.

**Fig 7:**
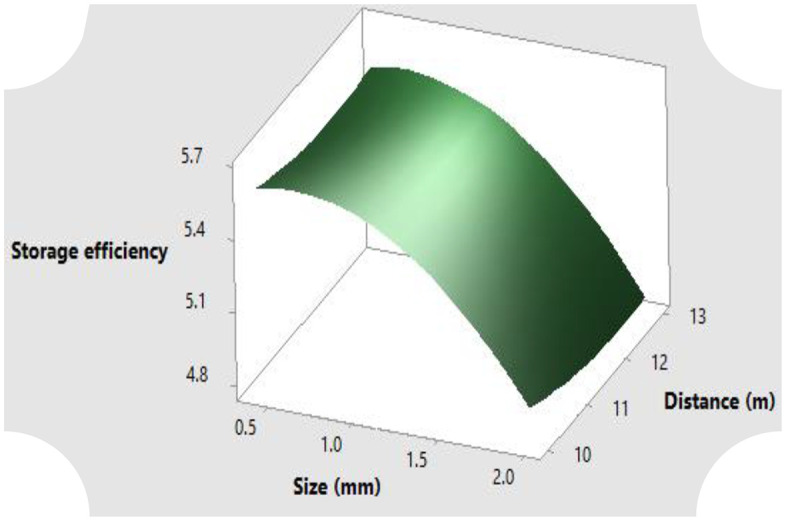
Storage efficiency response of the rosewood substrate to different particle sizes and distance away from fertigation source.

The surface response for the full quadratic model of the relationship between particle size and distance from reservoir on the parameters of hydrological properties of sawdust such as the moisture content both at saturation and after drainage, air capacity and storage efficiency are illustrated in Figs 4–7. Generally, the moisture content as well as the storage efficiency increases with finest of particle size, while the coarser the particle size, the more the air capacity.

### 3.6. Statistical importance of the Response Surface Methodology (RSM) for the prediction of the soil water retention, air capacity and storage efficiency of the substrate

The ANOVA results based on the RSM regression analysis that was performed to predict the hydro-physical parameters for the substrate are presented in S1–S4 Tables (Supplementary material). The p-value for the regression models for all the hydro-physical parameters (soil water content, air capacity and storage efficiency) were all significant (< 0.0001; S1–S4 Tables). This high level of significance indicates the strong effectiveness and efficiency of the RSM derived regression in predicting the hydro-physical responses (soil water content, air capacity and storage efficiency) based on the distance of the laterals from the water source containing nutrient solution and the sizes of the rose wood substrate used in the study, thus emphasizing the high reliability of the obtained modelling results. Furthermore, since there is only a 0.01% possibility that noise could account for an F-value this great, the models’ F-values of 48.29, 42.75, 40.07 and 32.83 for the water retention at saturation, after 24 hours of drainage (field capacity), air capacity and storage efficiency respectively all suggests that the model is significant. For this reason, the model input parameters; distance away from the water source containing nutrient solution, and the size of the substrates are important model terms in this instance. Moreso, the insignificance of the lack-of-fit F-values of 1.33, 1.20, 1.46 and 0.49 with p-values of 24.5, 31.5, 18.8, 81.3% for the water retention at saturation, after 24 hours of drainage (field capacity), air capacity and storage efficiency (S1–S4 Tables) respectively all indicate an excellent model for the prediction of all hydro-physical parameters output. Most notably, the statistical fitting yields an R^2^ value greater than 0.60, which is favorable for the fit of the regression model. In addition, the models’ ability to effectively anticipate the response (hydro-physical parameters) is demonstrated by the less than 0.2, that is, the difference between the adjusted and the predicted. Since the difference is less than 20% (0.2), it indicates high reliability and efficiency. For example, the difference was 1.35%, 1.46%, 1.52% and 2.09% (S1–S4 Tables) for the water retention at saturation, after 24 hours of drainage (field capacity), air capacity and storage efficiency. The R^2^ values of 68.64, 62.48, 60.95 and 62.48% obtained in the regression showed that more than 60% of the variability in the hydro-physical responses can be explained by the size of the substrate and distance away from the water source containing water solution. Further study is therefore recommended to be carried out to determine other input parameters, which could further explain the variability in the hydrological responses, for enhanced predictability.

### 3.7. Limitation of the study and recommendation for further study

This study only considered rosewood, which among other type of wood is commonly available in the region. However, for further study other wood types, which are commonly cut into smaller pieces in the sawmill present in the region should be tested. This is important in other to select the best substrate (wood source/type) that is most suitable for hydroponics, in order to complement agricultural production, and ultimately enhance agricultural productivity. Moreso, in this study the rosewood components like lignin, cellulose, nitrogen immobilization and hemi-cellulose contents were not considered, which are important for the substrate nutrient determination, as such were suggested for further study. Moreso, we recommend possible effective approach with proper solution, which could be applied to pretreat the rosewood for a reduced EC, for its efficiency and effectiveness in hydroponics to be optimized for crop production. In addition, other hydrology component like wilting point should be determined for rosewood, in order to complement the outcome of our findings. The estimation of the wilting point was not captured for rosewood in this study due to equipment limitation, particularly pressure plate

## 4. Conclusion

The study has been successful in characterizing the physical, chemical and hydrological characteristics of rosewood sawdust of different particle sizes for scientific knowledge, and to help in hydroponic consideration of rosewood sawdust as a potential growing medium in Omu Aran, Nigeria. The field capacity varied between 133 and 159%, while the total porosity varied between 72.6 and 75.9%, depending on the particle size of the rose wood. Though, the hydro-physical characteristics of rosewood sawdust from this study seems favourable for plant’s anchorage, roots’ development and aeration, the chemical parameter (EC in particular) may pose a detriment to crop grown on sawdust if not frequently monitored when used in hydroponics, hence, there will be need to select the best nutrient solution to neutralize the high EC. While the chemical characterization revealed that the substrate is mostly fit for agricultural production through the enhancement of the nutrient contents. This was tested with Cayenne pepper crop with successful germination achieved. The outcome from the study also indicated that water retention in the substrates was enhanced as a result of particle size. The established full quadratic model produced the highest R^2^ in the relationships between the sawdust particle sizes and distances for the moisture storage efficiency determination of the substrates.

## Supporting information

S1 Fig Map illustrating Omu Aran’s position in Kwara State, North Central Nigeria [51]. (DOCX)

S1 Table Response Surface Regression: moisture content at Saturation versus Size (mm), Distance (m). (DOCX)

S2 Table Response Surface Regression: moisture content after 24 hours versus Size (mm), Distance (m). (DOCX)

S3 Table Response Surface Regression: Air Capacity (AC) versus Size (mm), Distance (m). (DOCX)

S4 Table Response Surface Regression: Storage efficiency versus Size (mm), Distance (m). (DOCX)
