# Supplementary figures and images for: Hydro-physical and chemical suitability of rosewood sawdust as a hydroponic substrate under drip irrigation
Source: PLoS One. 2025 Nov 17;20(11):e0336497. doi: 10.1371/journal.pone.0336497 (PMC12622810; doi:10.1371/journal.pone.0336497)

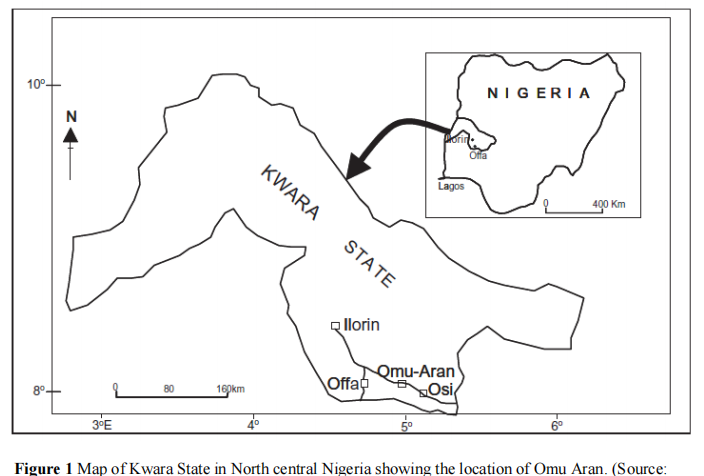


**S1 Fig.** Map illustrating Omu Aran's position in Kwara State, North Central Nigeria [51].

Supplement: S1 Fig — (DOCX) [file pone.0336497.s001.docx]
