# Supplementary material for: Hydro-physical and chemical suitability of rosewood sawdust as a hydroponic substrate under drip irrigation
Source: PLoS One. 2025 Nov 17;20(11):e0336497. doi: 10.1371/journal.pone.0336497 (PMC12622810; doi:10.1371/journal.pone.0336497)
Supplement: S1 Table — (DOCX) [file pone.0336497.s002.docx]

**S1 Table:** Response Surface Regression: moisture content at Saturation versus Size (mm), Distance (m)

Analysis of Variance

Source DF Adj SS Adj MS F-Value P-Value

Model 5 1.55849 0.311699 32.83 0.000

Linear 2 1.36083 0.680415 71.66 0.000

Size (mm) 1 0.37009 0.370088 38.98 0.000

Distance (m) 1 0.99074 0.990742 104.35 0.000

Square 2 0.07595 0.037973 4.00 0.022

Size (mm)*Size (mm) 1 0.04156 0.041560 4.38 0.040

Distance (m)*Distance (m) 1 0.03439 0.034387 3.62 0.061

2-Way Interaction 1 0.02576 0.025763 2.71 0.104

Size (mm)*Distance (m) 1 0.02576 0.025763 2.71 0.104

Error 75 0.71210 0.009495

Lack-of-Fit 6 0.02919 0.004865 0.49 0.813

Pure Error 69 0.68291 0.009897

Total 80 2.27059
