# Supplementary material for: Hydro-physical and chemical suitability of rosewood sawdust as a hydroponic substrate under drip irrigation
Source: PLoS One. 2025 Nov 17;20(11):e0336497. doi: 10.1371/journal.pone.0336497 (PMC12622810; doi:10.1371/journal.pone.0336497)
Supplement: S2 Table — (DOCX) [file pone.0336497.s003.docx]

**S2 Table:** Response Surface Regression: moisture content after 24 hours versus Size (mm), Distance (m)

Analysis of Variance

Source DF Adj SS Adj MS F-Value P-Value

Model 3 0.545798 0.181933 42.75 0.000

Linear 2 0.544133 0.272067 63.93 0.000

Size (mm) 1 0.536708 0.536708 126.11 0.000

Distance (m) 1 0.007425 0.007425 1.74 0.190

2-Way Interactions 1 0.000382 0.000382 0.09 0.765

Size (mm)*Distance (m) 1 0.000382 0.000382 0.09 0.765

Error 77 0.327714 0.004256

Lack-of-Fit 8 0.039882 0.004985 1.20 0.315

Pure Error 69 0.287831 0.004171

Total 80 0.873511
