# Supplementary material for: Hydro-physical and chemical suitability of rosewood sawdust as a hydroponic substrate under drip irrigation
Source: PLoS One. 2025 Nov 17;20(11):e0336497. doi: 10.1371/journal.pone.0336497 (PMC12622810; doi:10.1371/journal.pone.0336497)
Supplement: S3 Table — (DOCX) [file pone.0336497.s004.docx]

**S3 Table:** Response Surface Regression: Air Capacity (AC) versus Size (mm), Distance (m)

Analysis of Variance

Source DF Adj SS Adj MS F-Value P-Value

Model 3 0.98397 0.327991 40.07 0.000

Linear 2 0.85204 0.426018 52.04 0.000

Size (mm) 1 0.02541 0.025407 3.10 0.082

Distance (m) 1 0.82663 0.826628 100.98 0.000

2-Way Interactions 1 0.01987 0.019868 2.43 0.123

Size (mm)*Distance (m) 1 0.01987 0.019868 2.43 0.123

Error 77 0.63035 0.008186

Lack-of-Fit 8 0.09126 0.011407 1.46 0.188

Pure Error 69 0.53910 0.007813

Total 80 1.61433
