# Supplementary material for: Hydro-physical and chemical suitability of rosewood sawdust as a hydroponic substrate under drip irrigation
Source: PLoS One. 2025 Nov 17;20(11):e0336497. doi: 10.1371/journal.pone.0336497 (PMC12622810; doi:10.1371/journal.pone.0336497)
Supplement: S4 Table — (DOCX) [file pone.0336497.s005.docx]

**S4 Table:** Response Surface Regression: Storage efficiency versus Size (mm), Distance (m)

Analysis of Variance

Source DF Adj SS Adj MS F-Value P-Value

Model 3 6.7196 2.23986 42.75 0.000

Linear 2 6.6991 3.34954 63.93 0.000

Size (mm) 1 6.6077 6.60767 126.11 0.000

Distance (m) 1 0.0914 0.09141 1.74 0.190

2-Way Interactions 1 0.0047 0.00471 0.09 0.765

Size (mm)*Distance (m) 1 0.0047 0.00471 0.09 0.765

Error 77 4.0346 0.05240

Lack-of-Fit 8 0.4910 0.06138 1.20 0.315

Pure Error 69 3.5436 0.05136

Total 80 10.7542
